# Avian Influenza (H5N1) Susceptibility and Receptors in Dogs

**DOI:** 10.3201/eid1308.070393

**Published:** 2007-08

**Authors:** Riks Maas, Mirriam Tacken, Lisette Ruuls, Guus Koch, Eugene van Rooij, Norbert Stockhofe-Zurwieden

**Affiliations:** * Wageningen University and Research Centre, Lelystad, the Netherlands

**Keywords:** avian influenza, H5N1, dog, infection, receptor, dispatch

## Abstract

Inoculation of influenza (H5N1) into beagles resulted in virus excretion and rapid seroconversion with no disease. Binding studies that used labeled influenza (H5N1) showed virus attachment to higher and lower respiratory tract tissues. Thus, dogs that are subclinically infected with influenza (H5N1) may contribute to virus spread.

Avian influenza (H5N1) virus has been shown to be infectious not only for birds but also for humans and mammals such as mice, ferrets, and cats. Carnivorous mammals that are susceptible to subtype H5N1 may contribute to spread of the virus; shedding of influenza (H5N1) by pet carnivores may pose a risk to humans. Cats experimentally inoculated with influenza (H5N1) have been shown to be susceptible to infection and to shed virus (*1*). However, dogs’ susceptibility to this virus is unknown. Unpublished studies indicate that a substantial number of dogs tested in Thailand were positive for antibodies against H5N1 subtype (*2*). Recently, isolation of influenza (H5N1) virus from a dog in Thailand has been reported (*3*). We describe the susceptibility of specific pathogen–free (SPF) beagles to avian influenza (H5N1) and the presence of receptors for influenza (H5N1) in the respiratory tract of these dogs.

## The Study

To study the infectivity of avian influenza (H5N1) in dogs, we inoculated 3 SPF beagles (HsdCpb:DOBE; Harlan Nederland, Horst, the Netherlands), 16 weeks of age, with 10^6^ median tissue culture infectious doses of influenza (H5N1) (A/chicken/GxLA/1204/2004). Half of the dose (0.5 mL) was administered intranasally and the other 0.5mL intratracheally. Body temperature and health status of the dogs were monitored twice a day during the first 5 days after challenge and once a day thereafter. No major changes in body temperature and no clinical signs were noted.

Excretion of virus was monitored daily in swabs from rectum, oropharynx, and nose. The presence of influenza (H5N1) virus in these swabs was studied by inoculation into embryonated eggs and by real-time reverse transcription–PCR that targeted the matrix gene. For the PCR, we used the forward primer AI-M-F45 (5′-CTTCTAACCGAGGTCGAAACGTA-3′, reverse primer AI-M-R251 (5′-CACTGGGCACGGTGAGC-3′) and Taqman probe AI-M-Tqmn1 (5′-6FAM-CTCAAAGCCGAGATCGCGCAGA-XT-PH) (TIBMolBiol, Berlin, Germany). A calibration curve consisting of serial dilutions of a standard batch of influenza (H5N1) virus with a known median 50% egg infectious dose (EID_50_) titer was included in each PCR. One of the dogs shed virus for several days after challenge. In this dog, virus was demonstrated by PCR in nasal swabs taken on days 1 through 4 after challenge and by virus isolation in embryonated chicken eggs on days 2 and 3 after challenge (Table 1). Quantification by real-time PCR indicated that the amount of virus present in the nasal swabs corresponded to 2.0–3.2 log_10_ EID_50_.

**Table 1:** Virus detection in nasal swabs from dogs inoculated with avian influenza (H5N1)*

Dog	Days postinoculation										
	1			2			3			4	
	PCR†	Egg		PCR	Egg		PCR	Egg		PCR	Egg
1	10^3.2^	–		10^2.0^	+		10^2.9^	+		10^2.8^	–
2	–	–		–	–		–	–		–	–
3	–	–		–	–		–	–		–	–

*PCR, real-time PCR of nasal swabs from dogs; Egg, embryonated chicken eggs.; + virus detected; –, virus not detected. †After quantification by PCR, virus titers are expressed as 50% egg infectious dose.

Serum samples collected at days 7 and 14 after challenge were tested for antibodies against H5N1 subtype in an influenza A nucleoprotein-blocking ELISA as well as in a hemagglutination-inhibition assay. An antibody response against influenza (H5N1) was detectable in 1 dog at day 7 after challenge. In all dogs an antibody response against influenza (H5N1) was demonstrated in both assays at day 14 after challenge (Table 2). Postmortem examination on day 14 after virus challenge showed no gross pathologic or histopathologic changes in the respiratory tract and other organs. Considering the time of sampling after challenge, transient histopathologic changes may have occurred unnoticed.

**Table 2:** Antibody titers in serum of dogs inoculated with avian influenza (H5N1)*

Dog	Days postinoculation							
	0			7			14	
	ELISA	HI†		ELISA	HI†		ELISA	HI†
1	–	–		–	–		+	16
2	–	–		–	–		+	16
3	–	–		+	16		+	32

*Antibodies were measured in a nucleoprotein-blocking ELISA and in the hemagluttination-inhibition (HI) assay at different days postinoculation. +, antibodies detected; –, antibodies not detected. †HI titers are presented as the reciprocal to the highest serum dilutions completely inhibiting agglutination of chicken erythrocytes by influenza (H5N1) virus.

Influenza (H5N1) viruses bind with their hemagglutinin surface proteins to cell surface oligosaccharides terminating in sialic acid α2,3 galactose (SA2,3Gal) (*4*). In humans and cats, influenza (H5N1) virus predominantly attaches to the lower part of the respiratory tract where the SA2,3Gal receptors are present (*5*). To study the attachment pattern of influenza (H5N1) in the respiratory tract of dogs, we performed binding experiments with labeled influenza (H5N1) virus on formaldehyde-fixed and paraffin-embedded tissue sections. We collected tissues directly from a euthanized control dog of the same breed as that used for the infection experiment and fixed them in 4% buffered formaldehyde solution for several weeks. Virus labeling and histochemical examination were performed according to van Riel et al. (*5*). Briefly, influenza (H5N1) virus was grown in 10-day-old embryonated eggs and inactivated with formalin. After being purified by sucrose gradient centrifugation, the virus was labeled with fluorescein isothiocyanate (FITC). Staining was performed by incubating tissue sections with FITC-labeled virus and detecting the FITC-label with a peroxidase-labeled rabbit anti-FITC*.* To create a negative control, staining was performed without prior incubation with labeled virus or after prior incubation with FITC alone. No staining was observed in these negative controls. Chicken tissues were used as positive controls.

Strong virus binding to the epithelia of the chicken respiratory tract tissues was noted. Furthermore, as a specificity control, preincubation with *Maackia amurensis* lectin, which specifically binds to SA2,3Gal–terminated oligosaccharides, was performed. Moderate particulate binding of influenza (H5N1) to canine nasal mucosa, tracheal epithelium, and alveoli was observed. Strong multifocal binding was observed in bronchial epithelium (Figure). This staining pattern was also found after binding of FITC-labeled *M. amurensis* lectin to canine respiratory tract tissues. Virus binding could be blocked with unlabeled *M. amurensis* lectin.

**Figure:**
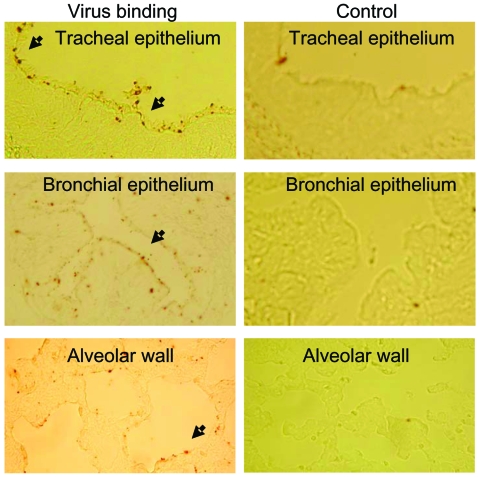
Binding of fluorescein isothiocyanate–labeled influenza (H5N1) virus to formaldehyde-fixed, paraffin-embedded tissue slides of dog respiratory tract tissues. Left panel shows binding of virus (arrow). Right panel shows blocking of virus binding by competitive binding of *Maackia amurensis* lectin to sialic acid α2,3 galactose.

## Conclusions

Our results demonstrate that dogs are susceptible to infection with avian influenza (H5N1) virus and can shed virus from the nose without showing apparent signs of disease. Moreover, receptors for avian (H5N1) virus are present not only in the lower part of the respiratory tract of dogs but also in their trachea and nose, which are potential portals of entry for the virus.

Influenza virus infection of dogs was first reported in 2004 (*6*). Influenza (H3N8) of equine origin caused outbreaks in greyhounds in Florida and has since been found in dogs in >20 US states (*7*). The course of experimental infection of SPF dogs with subtype H5N1 resembles that of the experimental infection of dogs with the subtype H3N8 (*6*): all dogs seroconverted, and some excreted virus without obvious disease. In contrast to the experimental outcomes, natural infections with influenza (H3N8) resulted in serious illness, death, and widespread infection for dogs. This finding warrants special attention to the potential course of avian influenza (H5N1) infection in dogs. Therefore, dogs’ contact with birds and poultry should be avoided in areas with influenza (H5N1) outbreaks to prevent possible spread of virus and human exposure to influenza (H5N1) virus that might have been adapted to mammals.
